# Effect of Intravenous Paracetamol on Opioid Consumption in Multimodal Analgesia After Lumbar Disc Surgery: A Meta-Analysis of Randomized Controlled Trials

**DOI:** 10.3389/fphar.2022.860106

**Published:** 2022-05-23

**Authors:** Feng Yin, Xiu-Hong Wang, Fei Liu

**Affiliations:** Department of Anesthesiology, West China Hospital, Sichuan University, Chengdu, China

**Keywords:** paracetamol, intravenous, multimodal analgesia, lumbar disc, opioid consumption

## Abstract

**Background:** Intravenous paracetamol, as an adjunct to multimodal analgesia, has been shown to successfully reduce opioid consumption after joint arthroplasty, abdominal surgery, and caesarean delivery. However, there are limited data on the opioid-sparing effect of intravenous paracetamol on lumbar disc surgery.

**Objectives:** The aim of this study was to investigate the effectiveness and safety of intravenous paracetamol for reducing opioid consumption in lumbar disc surgery. The primary outcome was cumulative opioid consumption within 24 h postoperatively.

**Method:** We followed the PRISMA-P guidelines and used GRADE to assess the quality of evidence. The review was registered in PROSPERO under the registration number CRD42021288168. Two reviewers conducted electronic searches in PubMed, Embase, Cochrane Central Register of Controlled Trials (CENTRAL), Scopus, and Web of Science (Clarivate Analytics). Randomized controlled trials (RCTs) that compared the postoperative opioid consumption of intravenous paracetamol with placebo in lumbar discectomy were included.

**Results:** Five trials comprising a total of 271 patients were included. The overall opioid consumption within 24 h postoperatively was reduced [mean difference (MD), −10.61 (95% CI, −16.00 to −5.22) mg, *p* = 0.0001, I^2^ = 90%] in patients with intravenous paracetamol. Intravenous paracetamol significantly reduced the postoperative pain scores at 1 h [MD, −2.37 (95%CI, −3.81 to −0.94), *p* = 0.001, I^2^ = 82%], 2 h [MD, −3.17 (95%CI, −3.85 to −2.48), *p* < 0.00001, I^2^ = 38%], 6 h [MD, −1.75 (95%CI, −3.10 to −0.40), *p* = 0.01], 12 h [MD, −0.96 (95%CI, −1.77 to −0.15), *p* = 0.02], and 24 h [MD, −0.97 (95%CI, −1.67 to −0.27), *p* = 0.006] compared with the placebo. There were no differences in postoperative adverse effects.

**Conclusion:** Intravenous paracetamol reduced postoperative opioid consumption and decreased postoperative pain scores without increasing adverse effects. The overall GRADE quality of the evidence was rated as low to moderate. Intravenous paracetamol appears to be an applicable option as an important part of multimodal analgesia for postoperative analgesia after lumbar disc surgery.

**Systematic Review Registration:** https://www.crd.york.ac.uk/prospero/, CRD42021288168.

## Introduction

Lumbar disc surgery, as the most commonly performed surgical spine procedure (Gibson and Waddell, 2007), is associated with moderate to severe back and radicular pain postoperatively (Kesimci et al., 2011), which could result in delayed rehabilitation (Goudas et al., 1999), increased morbidity, prolonged length of stay, and high cost and hospital readmission rate (Nielsen and Steele, 2002). It is essential to manage pain effectively with minimal adverse effects for enhanced functional recovery and reduced postoperative morbidity (Kehlet and Dahl, 2003; White, 2005).

Opioids, widely used for treating postoperative pain, are associated with adverse effects such as nausea, vomiting, dizziness, urinary retention, itching, and respiratory depression (He et al., 2020; Brixel et al., 2021). Multimodal analgesia (MMA) regimens could reduce opioid consumption and opioid-related complications (Hebl et al., 2008; Tayrose et al., 2013), which have been strongly recommended to alleviate postoperative pain in the Clinical Practice Guideline (Chou et al., 2016) and the American Society of Anesthesiologists (ASA) (Practice guidelines for acute pain, 2012).

Paracetamol (acetaminophen) is an important component of MMA regimens and is generally recognized as a safe and effective medication in treating postoperative pain with a favorable adverse effect profile (Kesimci et al., 2011; Lachiewicz, 2013). Paracetamol exerts a central analgesic effect through the inhibition of COX-2 activity and the activation of descending serotonergic pathways (Pickering et al., 2006). The intravenous (IV) formulation of paracetamol was approved by the United States Food and Drug Administration (FDA) in 2010 for the management of moderate to severe pain with adjunctive opioid medication (Lachiewicz, 2013). The analgesic effect of IV paracetamol including decreased opioid consumption and pain scores varied depending on the surgery. Some meta-analyses demonstrated that IV paracetamol decreased rescue opioid consumption and pain scores in patients undergoing total knee and hip arthroplasty and bariatric surgery (Liang et al., 2017; Yang et al., 2017; Lee et al., 2019; Ghaffarpasand et al., 2020). However, it was found that IV paracetamol could reduce opioid consumption, but it did not contribute to a decrease in the average pain scores for total joint arthroplasty, as shown in a meta-analysis by Guo et al. (2018). Furthermore, it was found that IV paracetamol could not significantly decrease narcotic consumption and pain scores compared with the placebo in adult patients undergoing abdominal surgery, as shown in a meta-analysis by Blank et al. (2018). The utility of IV paracetamol for pain management after lumbar disc surgery is relatively unclear as randomized controlled trials on the topic have achieved mixed results.

As both the use of lumbar disc surgery and concerns over opioids are growing topics of interest, and doubts remain regarding the benefit of IV paracetamol in lumbar discectomy, the opioid-sparing effect of IV paracetamol in patients undergoing lumbar disc surgery is an increasingly important question. To our knowledge, there was no meta-analysis systematically evaluating the available evidence of IV paracetamol for pain management after lumbar disc surgery. Therefore, we conducted a systematic review and meta-analysis to update the existing evidence and gain further insight into the opioid-sparing effects and safety of IV paracetamol in patients undergoing lumbar discectomy.

## Methods

### Data Sources and Searches

We systematically searched PubMed, Embase, Cochrane Central Register of Controlled Trials (CENTRAL), Scopus, and Web of Science (Clarivate Analytics) from the database inception to October 29, 2021, with an update performed before submission for publication. We also searched the grey literature (including reports, conferences, workshop proceedings, and ongoing trials) using the clinical trials registry (www.clinicaltrials.gov) and Google Scholar. We checked the reference lists of all included studies to identify any studies missed from the original search. No language or publication date restrictions were applied.

The following search strategy was used for PubMed: (Acetaminophen [mh] OR Acetaminophen [tiab] OR Hydroxyacetanilide [tiab] OR APAP [tiab] OR p-Acetamidophenol [tiab] OR p-Hydroxyacetanilide [tiab] OR Paracetamol [tiab] OR N-(4-Hydroxyphenyl)acetanilide [tiab] OR Acetamidophenol [tiab] OR N-Acetyl-p-aminophenol [tiab] OR Acephen [tiab] OR Acetaco [tiab] OR Tylenol [tiab] OR Anacin-3 [tiab] OR Anacin 3 [tiab] OR Anacin3 [tiab] OR Datril [tiab] OR Panadol [tiab] OR Acamol [tiab] OR Algotropyl [tiab]) AND (IV [tiab] OR intravenous [tiab]) AND lumbar [tiab] AND (randomized controlled trial [pt] OR controlled clinical trial [pt] OR randomized [tiab] OR placebo [tiab] OR randomly [tiab] OR trial [tiab] OR groups [tiab]). The search strategies used are available in Supplementary Table S1.

### Eligibility Criteria

Studies were eligible if (Gibson and Waddell, 2007), population: patients undergoing lumbar disc surgery (Kesimci et al., 2011); intervention: perioperative administration of IV paracetamol for postoperative analgesia (Goudas et al., 1999); control: administration of placebo (Nielsen and Steele, 2002); outcomes: eligible studies must report at least one of predetermined outcomes (Kehlet and Dahl, 2003); and design: randomized controlled trials (RCTs). No language, sample size, or date of publication restrictions was applied. In studies with several control groups, the data were assessed between IV paracetamol and the placebo. Exclusion criteria were as follows (Gibson and Waddell, 2007): duplication of research literature (Kesimci et al., 2011); results and complete study details were unavailable after contacting the authors (Goudas et al., 1999); and unextractable data.

### Protocol and Registration

This systematic review followed the Preferred Reporting Items for Systematic Reviews and Meta-Analyses Protocols (PRISMA-P) recommendations (Moher et al., 2009). The protocol was also registered with the International Prospective Register of Systematic Reviews (PROSPERO, https://www.crd.york.ac.uk/prospero/, CRD42021288168).

### Study Selection and Data Collection

Two reviewers (FY and X-HW) independently screened the retrieved titles and abstracts for potential inclusion, reviewed the complete text of potentially eligible studies, and extracted data using a uniform data extraction form specifically developed for this review. Extracted data were entered in a standardized format into Microsoft Excel 2019 (Microsoft Corporation, United States). The following data were extracted from each study: first author, year of publication, number of enrolled patients, distribution in both groups, type of surgery and anesthesia, protocol for the paracetamol and placebo groups, peri-operative analgesia regimens, and study outcomes. We contacted the corresponding authors if relevant outcome data were missing. Any disagreement was resolved through discussion between the two reviewers or mediated by a third reviewer (FL).

### Primary and Secondary Outcome

The primary outcome was the total morphine consumption at 24 h postoperatively presented in milligrams (mg). Secondary outcomes were the pain grades at 1, 2, 6, 12, and 24 h postoperatively [only pain grades scored as 0 to 10 or 0 to 100 were included as these could be converted to a numeric rating scale (NRS), NRS 0 to 10, 0 = no pain and 10 = maximum pain], the incidence of postoperative nausea and vomiting (PONV), and urinary retention.

### Risk of Bias

Two reviewers (FY and X-HW) assessed the risk of bias in the included studies independently and in duplicate using tools developed by the Cochrane Collaboration risk of bias tool (Higgins et al., 2021). Studies were categorized into high, low, or unclear risk of bias according to the following predefined domains: random sequence generation (selection bias), allocation concealment (selection bias), blinding of participants and personnel (performance bias), blinding of outcome assessment (detection bias), incomplete outcome data (attrition bias), selective reporting (including 1) selective non-reporting of results, where results for some of the analyzed outcomes are selectively omitted from a published report; 2) selective under-reporting of data, where results for some outcomes are selectively reported with inadequate details for the data to be included in a meta-analysis; and 3) bias in selection of the reported result, where a result has been selected for reporting by the study authors, on the basis of the results, from multiple measurements or analyses that have been generated for the outcome domain), and other potential sources of bias, for example, the inaccurate data which were extracted from the included studies could produce extractor bias; improper and incomplete quality evaluation of the included studies may lead to a bias in scoring the study quality; and conflicts of interest may lead to a bias in the effect estimates from a trial by several mechanisms as the following: first, if those who recruited participants to participate in the trial had important conflicts of interest and the allocation sequence was not concealed, they may be more likely to subvert the distribution process to produce an unbalanced intervention group to support their preferred intervention. Similarly, researchers with important conflicts of interest may decide to exclude some patients who did not respond to the experimental intervention from the analysis, which would result in a bias due to the lack of outcome data. In addition, selective reporting of favorable results may be closely related to conflicts of interest as selective reporting of specific outcome measurements or selective reporting of specific analyses. The overall risk of bias for each included study was judged as “low” if the risk of bias was low in all domains, “unclear” if the risk of bias was unclear in at least one domain and with no high risk of bias in the domain, or “high” if the risk of bias was high in at least one domain. Each study was compared for consistency, with any disagreement resolved by discussion between the two reviewers or mediated by a third reviewer (FL).

### Quality of Evidence

The overall quality of evidence for each outcome was assessed by the Grading of Recommendations, Assessment, Development and Evaluations (GRADE) system using the GRADEpro Guideline Development Tool (Software) (Guyatt et al., 2008). The meta-analysis of RCTs began as high quality evidence and they were rated down based on the following five categories: risk of bias, imprecision, inconsistency, indirectness, and publication bias. The quality of evidence was categorized as high, moderate, low, or very low (FY and X-HW).

### Statistical Analysis

Studies with more than two treatment groups were handled as separate study results. We presented mean differences (MDs) for continuous data and relative risks (RRs) for dichotomous outcomes including corresponding 95% confidence intervals (CIs). To calculate the relative risk, the total number of participants in each group and those with the event of interest were extracted from each research. We performed a subgroup analysis according to the timing of administrating the study medication (before the anesthesia induction vs. before the end of the operation vs. after surgery) and the dose of paracetamol (single-dose or repeated-dose). Heterogeneity was assessed between studies using I^2^ statistics, with considerable heterogeneity predefined as I^2^ >50% (Higgins et al., 2003). A random-effects model was created to consider the clinical and methodological diversity between studies where heterogeneity existed across studies (Fleiss, 1993). All statistical analyses and meta-analyses were performed using RevMan software (Review Manager, version 5.4; the Nordic Cochrane Centre, the Cochrane Collaboration, Copenhagen, Denmark). A two-sided *p* value of less than 0.05 was considered statistically significant.

## Results

### Study Selection

**FIGURE 1:**
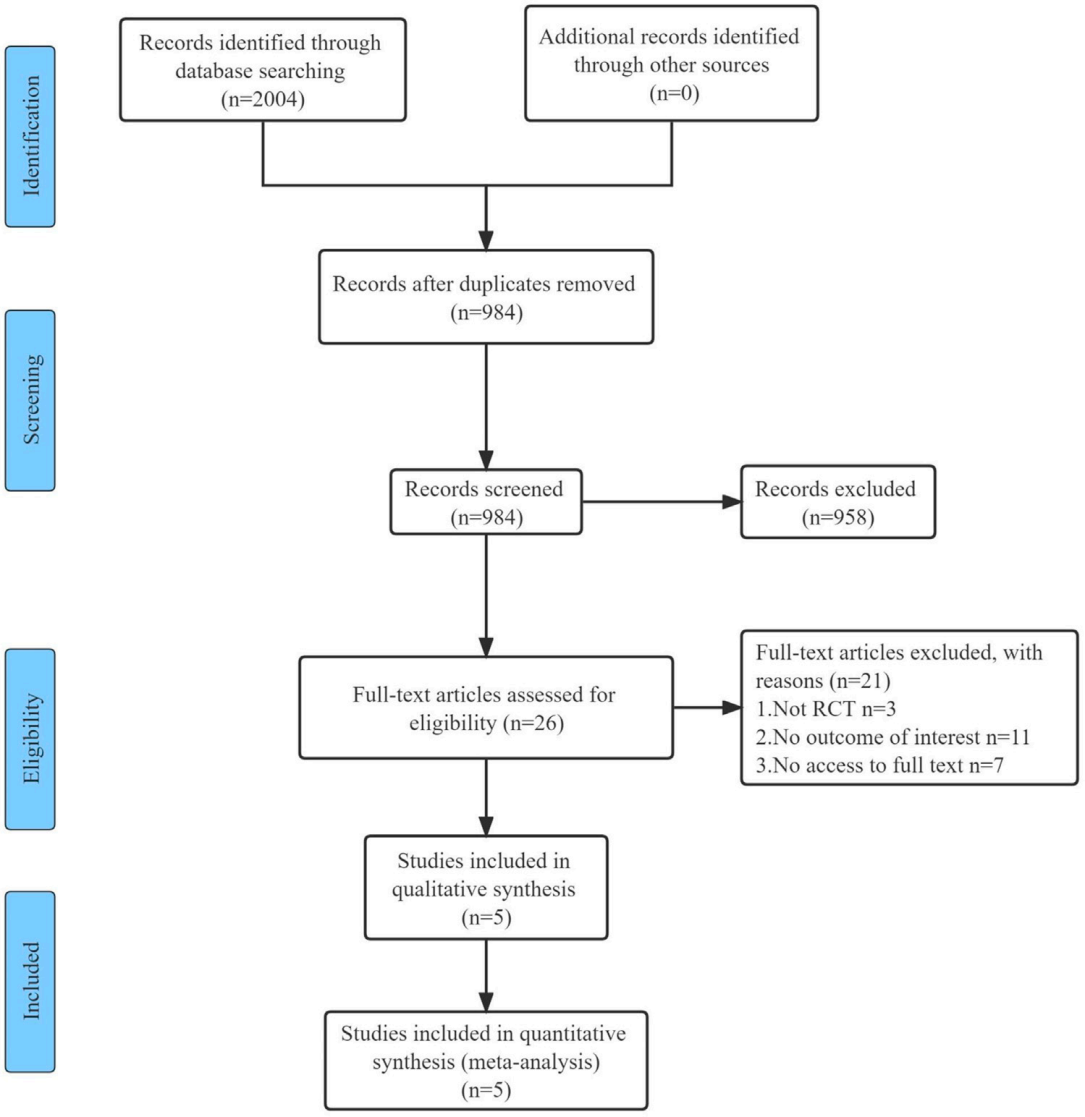
PRISMA flow diagram of the trial selection. RCT, randomized controlled trial.

The initial electronic search yielded 2,004 studies, of which 984 were screened and 26 were potentially eligible for full-text review. After duplicate and ineligible studies were removed, five RCTs with a total of 271 participants were finally included in our systematic review and meta-analysis. A detailed summary of the search performed is presented in the PRISMA Flowchart (Figure 1).

### Study Characteristics and Participants

Table 1 presents the characteristics of included studies. All works of research were single-center RCTs with a sample size of 40 to 60 patients. All trials but one (Shimia et al., 2014), without describing the anesthesia method, had lumbar disc surgery administering general anesthesia. Because Toygar et al. (2008) compared the analgesic effect of different times of IV paracetamol used (group I used IV paracetamol before the anesthesia induction, group II used before the end of surgery, and group III used placebo) and all the groups were eligible for our review and meta-analysis, we assessed data between two paracetamol groups and the placebo group separately. With the exception of one study, simultaneously comparing the preemptive analgesic effect of IV paracetamol used before the end of the surgery with before anesthesia induction (Toygar et al., 2008), three of the five studies used IV paracetamol before the end of surgery for postoperative analgesia (Uzun et al., 2010; Shimia et al., 2014; Akbas et al., 2021), and one study used after the surgery (Korkmaz Dilmen et al., 2010). Among all the included studies, only one trial focused on the postoperative analgesic effects of the repeated dose of IV paracetamol (Korkmaz Dilmen et al., 2010) while other trials concentrated on the efficacy of a single-dose of IV paracetamol (Toygar et al., 2008; Uzun et al., 2010; Shimia et al., 2014; Akbas et al., 2021). The control groups varied across the studies, consisting of groups with placebo or comparative analgesic medications, and we assessed data only between IV paracetamol and placebo in studies with several control groups. The dose and time of IV paracetamol used and the details of the control groups in each study are described in Table 1.

**TABLE 1:** Overview of analyzed studies.

Author (year)	Study group (n)	Anesthesia method	Study protocol	Peri-operative analgesia	Primary outcome (opioid consumption)	Secondary outcome (pain intensity)	Adverse events
Akbas et al. (2021)	Paracetamol	GA	Paracetamol (single dose: 1 g IV paracetamol before the end of surgery)	Intra-fentanyl; Post-PCA (morphine)	Morphine consumption in 24 h	VAS at 1, 2, 12, 24 h	PONV, pruritus, urinary retention
	Ibuprofen		Ibuprofen (800 mg of IV ibuprofen before the end of surgery)				
	Placebo		Placebo (250 ml normal saline before the end of surgery)				
Korkmaz Dilmen et al. (2010)	Paracetamol	GA	Paracetamol (repeated dose: 1 g IV paracetamol after surgery and repeated every 6 h)	Intra-remifentanil; Post-PCA (morphine)	Morphine consumption in 1, 2, 6, 12, 24 h	VAS at 1, 2, 6, 12, 24 h	PONV, rash, pruritus, urinary retention
	Metamizo		Metamizo (1 g IV metamizo started after surgery and repeated every 6 h)				
	Lornoxicam		Lornoxicam (8 mg IV lornoxicam started after surgery and repeated every 12 h)				
	Placebo		Placebo (isotonic saline)				
Shimia et al. (2014)	Paracetamol	-	Paracetamol (single dose: 1 g IV paracetamol within the last 20 min of surgery)	not mentioned	Morphine consumption in 24 h	VAS at 1, 6, 12, 18, 24 h	PONV, dizziness, constipation, urinary retention
	Placebo		Placebo (100 ml 0.9% sodium chloride within the last 20 min of surgery)				
Toygar et al. (2008)	Paracetamol I	GA	Paracetamol I (single dose: 1 g IV paracetamol 15 min before anesthesia induction)	Intra-remifentanil; Post -PCA (morphine)	Morphine consumption in 24 h	VAS at 0, 1, 2, 3, 6, 12, 24 h	PONV, urinary retention
	Paracetamol II		Paracetamol II (single dose: 1 g IV paracetamol started 15 min before the end of surgery)				
	Placebo, Korkmaz Dilmen et al. (2010)		Placebo (sodium chloride)				
Uzun et al. (2010)	Paracetamol	GA	Paracetamol (single dose: 1 g paracetamol IV at the end of the operation)	Intra-fentanyl; Post-PCA (morphine)	Morphine consumption in 24 h	VAS at 15 min, 30 min, 1, 2, 6, 24 h	PONV, shivering, urinary retention
	Paracetamol-metamizole		Paracetamol-metamizole (1 g paracetamol IV at the end of the operation and 1 g metamizole IV during the skin closure)				
	Placebo		Placebo (sodium chloride)				

GA, general anesthesia; IV, intravenous; PCA, patient-controlled analgesia; VAS, visual analog scale; PONV, postoperative nausea and vomiting.

The primary and secondary outcomes with more detailed descriptions of each individual study are presented in Table 1. The consumption of morphine was assessed at 24 h postoperatively and the pain scores were evaluated at different time points ranging from 1 h to 24 h postoperatively. There were three studies reporting pruritus (Korkmaz Dilmen et al., 2010; Akbas et al., 2021), and other adverse effects such as rash (Korkmaz Dilmen et al., 2010), dizziness (Shimia et al., 2014), constipation (Shimia et al., 2014), and shivering (Uzun et al., 2010) were reported in one study separately. The follow-up period for the outcomes in each study was 24 h.

### Risk of Bias Within and Across Studies and the Quality of Evidence

**FIGURE 2:**
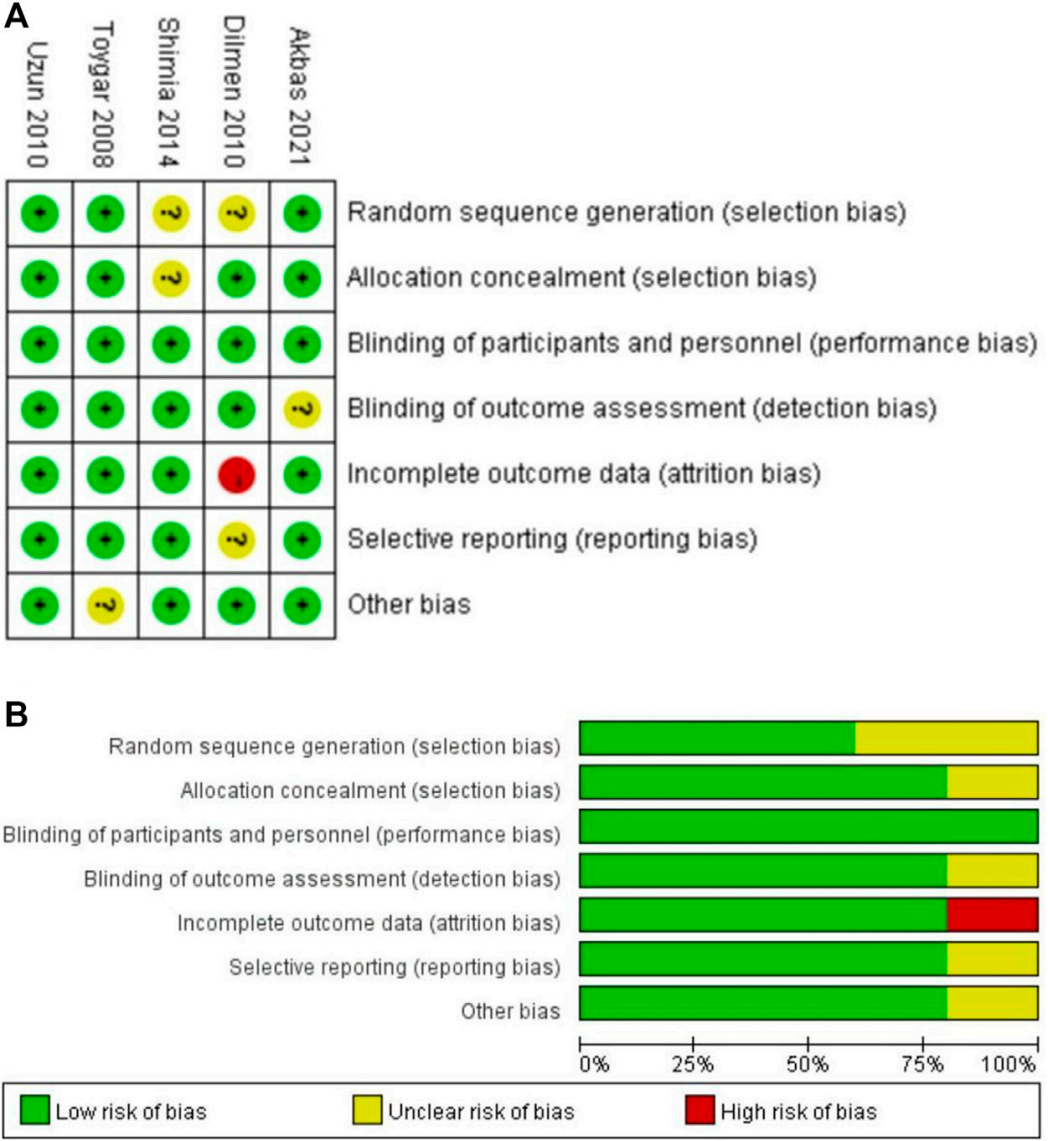
Risk of bias assessment. **(A)** Risk of the bias summary. **(B)** Risk of the bias graph. The green and plus signs indicate low risk, the red and minus signs indicate high risk, and the yellow and question marks indicate uncertain risk.

Among all studies, random sequence generation was shown in three studies (60%) (Toygar et al., 2008; Uzun et al., 2010; Akbas et al., 2021), allocation concealment in four studies (80%) (Toygar et al., 2008; Uzun et al., 2010; Akbas et al., 2021; Korkmaz Dilmen et al., 2010), blinding of participants and personnel in five studies (100%) (Shimia et al., 2014; Toygar et al., 2008; Uzun et al., 2010; Akbas et al., 2021; Korkmaz Dilmen et al., 2010), and blinding of the outcome assessment in four studies (80%) (Shimia et al., 2014; Toygar et al., 2008; Uzun et al., 2010; Korkmaz Dilmen et al., 2010). Incomplete outcome data were adequately explained in four studies (80%) (Shimia et al., 2014; Toygar et al., 2008; Uzun et al., 2010; Akbas et al., 2021), and one study (20%) (Korkmaz Dilmen et al., 2010)had selective reporting of outcomes. According to the Cochrane Collaboration’s risk of bias tool, we judged the risk of bias in all domains to be low in only one study (Uzun et al., 2010), unclear in three studies (Shimia et al., 2014; Toygar et al., 2008; Akbas et al., 2021), while one trial had a high risk of bias (Korkmaz Dilmen et al., 2010) (Figure 2). Domains accounting for the most judgments of unclear or high risk of bias were adjusted for the random sequence generation [three studies (Korkmaz Dilmen et al., 2010; Shimia et al., 2014; Ng et al., 2019)], allocation concealment [two studies (Shimia et al., 2014; Ng et al., 2019)], and selective reporting [two studies (Korkmaz Dilmen et al., 2010; Xuan et al., 2021)]. Domains accounting for the most judgments of unclear or high risk of bias were adjusted for the random sequence generation [two studies (Korkmaz Dilmen et al., 2010; Shimia et al., 2014), allocation concealment (one study (Shimia et al., 2014)], and selective reporting [one study (Korkmaz Dilmen et al., 2010)]. According to the Cochrane Collaboration risk of bias tool (Higgins et al., 2021), we compared the pre-specified content in a trial protocol with available content in the final trial report to access the evidence of selective non-reporting and under-reporting of results in randomized trials. Furthermore, we examined prudently the multiple outcome measurements and analyses of the data to determine whether the results being assessed and reported were likely to have been selected based on preferred results. In our analysis, Korkmaz Dilmen et al. (2010) indicated that the Ramsay score would be assessed in the outcome section, however, they did not describe the difference in the Ramsay score between the two groups finally. We tried to contact the author by email, but did not get a reply. Thus, we considered that this study had a selective reporting bias of outcomes. For the potential sources of bias in our analysis, we have obtained the original text of Toygar et al. (2008) that was presented in Turkish and we translated it through a translation software. However, there may still be some inaccurate data because of language limitations, which then results in extractor bias. We tried several methods recommended by the Cochrane Collaboration risk of bias tool (Higgins et al., 2021) to explore whether there were conflicts of interest in the included research. First, we accessed the trial protocol and statistical analysis plan to determine which outcomes and analyses were pre-specified for trials with relevant conflicts of interest. Furthermore, we examined conflicts of interest of trial co-authors and any commercial collaborators, and the lead and corresponding authors based on the information reported in the trial publication and the author declaration. Third, we searched for conflicts of interest data from the disclosure in other publications by the authors, the trial protocol, the clinical study report, and public conflicts of interest registries to avoid undisclosed conflicts of interest. Moreover, we searched ClinicalTrials.gov and conflicts of interest declarations in a few previous publications by the study authors in trials with unclear funding sources and no declaration of conflicts of interest from the lead or corresponding authors. However, we did not find any conflicts of interest among the co-authors, funding providers, or commercial collaborators in the including trials. The statistical analysis of publication bias for the primary outcome was performed using Stata 13.0 (Stata Corp, College Station, Texas, United States), and *p* values were generated from Begg’s test and Egger’s test as *p* < 0.10 was considered significant. There was no publication bias in this analysis with *p* = 1.0 by Begg’s test and *p* = 0.844 by Egger’s test. The funnel diagram of the publication bias has been uploaded as Supplementary Figure S1. The GRADE assessment demonstrated a low to moderate level of evidence for all outcomes (Supplementary Table S2).

### Pooled Results of the Included Studies

#### Primary Outcome: Total Morphine Consumption During 24 h Postoperatively

**FIGURE 3:**
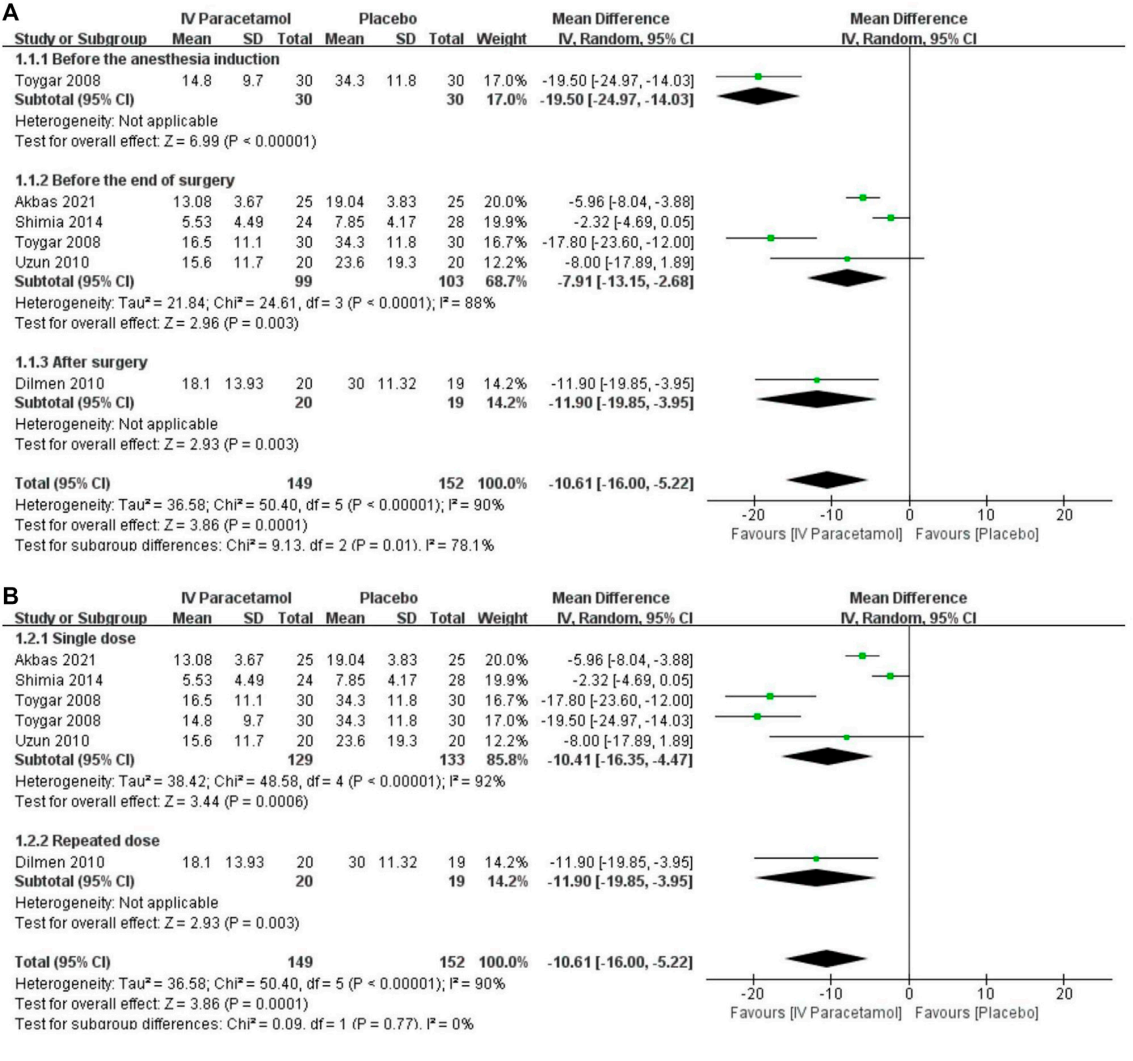
Forest plot showing the 24-h morphine consumption postoperatively (mg) with subgroup analyses presented according to the **(A)** time and **(B)** dose of intravenous paracetamol used. IV, intravenous. SD, standard deviation. CI, confidence interval.

Five studies reported total morphine consumption within 24 h after lumbar discectomy (Shimia et al., 2014; Toygar et al., 2008; Uzun et al., 2010; Akbas et al., 2021; Korkmaz Dilmen et al., 2010), and all the data were available as mean ± SD and were included in the meta-analysis. One study comparing different times of IV paracetamol used with the placebo was included in the analysis separately (Toygar et al., 2008). IV paracetamol had significantly less morphine consumption at 24 h compared with the placebo [MD, −10.61 (95% CI, −16.00 to −5.22) mg, *p* = 0.0001, I^2^ = 90%]. Heterogeneity existed between the five studies according to the results of the meta-analysis and data were analyzed with a random-effects model. Subgroup analyses according to the timing of paracetamol used and the way paracetamol was administrated were performed to assess the reason for heterogeneity. For both IV paracetamol used before the anesthesia induction [MD, −19.50 (95% CI, −24.97 to −14.03) mg, *p* < 0.00001] (Toygar et al., 2008), before the end of surgery [MD, −7.91 (95% CI, −13.15 to −2.68) mg, *p* = 0.003] (Shimia et al., 2014; Toygar et al., 2008; Uzun et al., 2010; Akbas et al., 2021), and after surgery [MD, −11.90 (95% CI, −19.85 to −3.95) mg, *p* = 0.003] (Korkmaz Dilmen et al., 2010), the total morphine consumption during 24 h postoperatively in the IV paracetamol group was lower than that in the placebo group (Figure 3A). Heterogeneity existed in both patients receiving single-dose IV paracetamol [MD, −10.41 (95% CI, −16.35 to −4.47) mg, *p* = 0.0006] (Toygar et al., 2008; Uzun et al., 2010; Shimia et al., 2014; Akbas et al., 2021) and patients having repeated-dose IV paracetamol [MD, −11.90 (95% CI, −19.85 to −3.95), *p* = 0.003] (Korkmaz Dilmen et al., 2010) (Figure 3B). The overall GRADE quality of evidence was rated as moderate because of heterogeneity in the pooled estimate (Supplementary Table S2).

### Secondary Outcomes: Pain Scores

**FIGURE 4:**
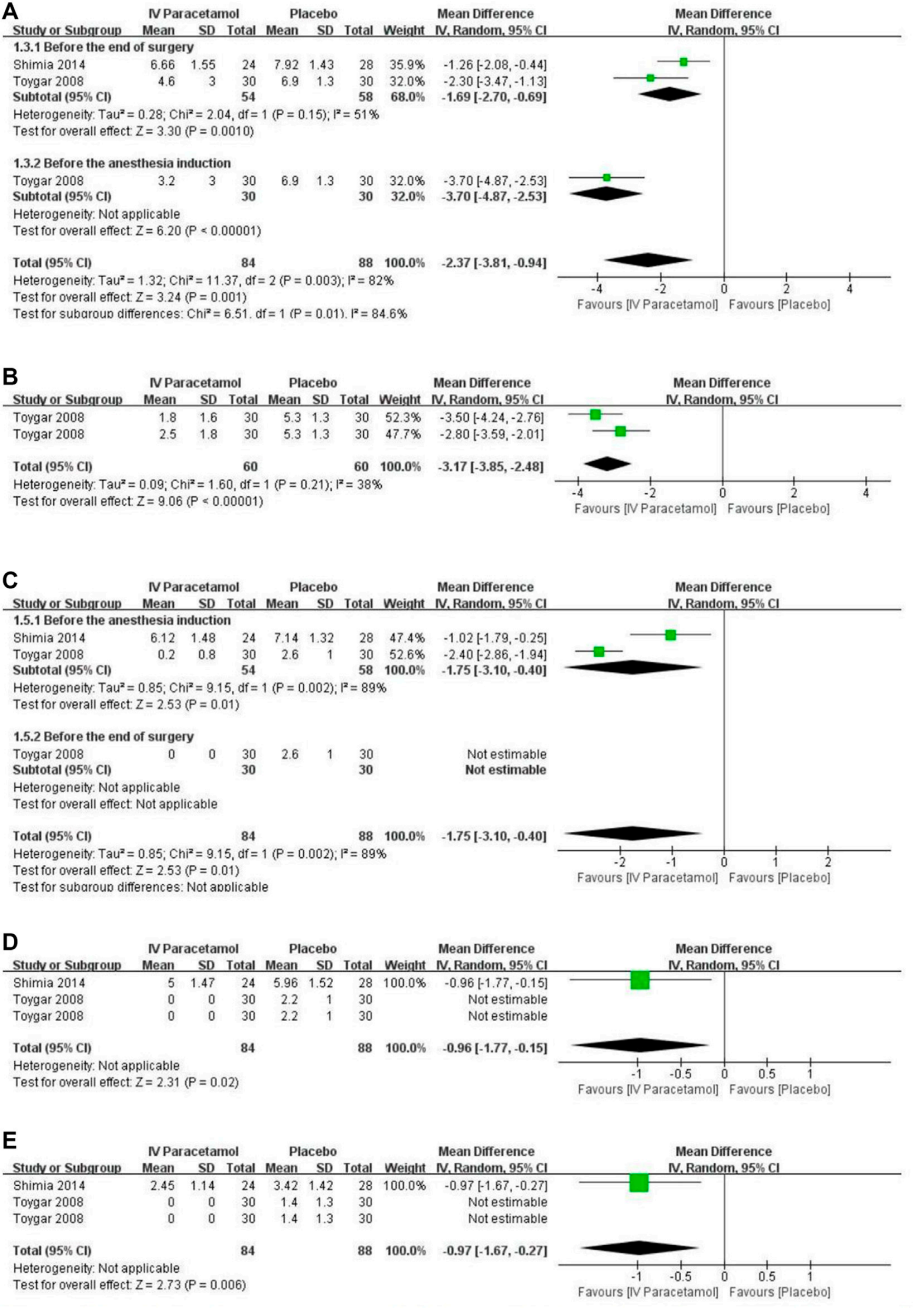
Forest plot for pain scores at **(A)** 1 h, **(B)** 2 h, **(C)** 6 h, **(D)** 12 h, and **(E)** 24 h. IV, intravenous. SD, standard deviation. CI, confidence interval.

Based on the data from two RCTs including 142 participants (Shimia et al., 2014; Toygar et al., 2008), pain scores were presented as mean ± SD and included in the analysis. Of these data, pain scores were significantly lower in the IV paracetamol group postoperatively at 1 h [MD, −2.37 (95%CI, −3.81 to −0.94), *p* = 0.001], 2 h [MD, −3.17 (95%CI, −3.85 to −2.48), *p* < 0.00001] (Figure 4B), 6 h [MD, −1.75 (95%CI, −3.10 to −0.40), *p* = 0.01], 12 h [MD, −0.96 (95%CI, −1.77 to −0.15), *p* = 0.02], and 24 h [MD, −0.97 (95%CI, −1.67 to −0.27), *p* = 0.006] compared with the placebo group (Figure 4). At 1 h after surgery (Figure 4A), two studies were included (Shimia et al., 2014; Toygar et al., 2008) and heterogeneity existed between the studies according to the results of the meta-analysis (I^2^ = 82%). Data were analyzed with a random-effects model. There was a subgroup effect in terms of the time IV paracetamol was used (*p* = 0.01). With regard to the subgroup analysis, when paracetamol was used before the end of surgery [MD, −1.69 (95% CI, −2.70 to −0.69), *p* = 0.001) and before the anesthesia induction [MD, −3.70 (95% CI, −4.87 to −2.53), *p* < 0.00001) showed a statistically significant difference. Two RCTs were included in the meta-analysis at 6 h (Shimia et al., 2014; Toygar et al., 2008) (Figure 4C), and heterogeneity was observed (I^2^ = 89%) with no applicable subgroup effect. In the time of 12 h (Figure 4D) and 24 h (Figure 4E), no applicable heterogeneity existed between the included two studies (Toygar et al., 2008; Shimia et al., 2014). There were insufficient data to conduct a subgroup analysis for the type of paracetamol used. The GRADE of evidence was rated as moderate because of existing risks of bias (Supplementary Table S2).

### Secondary Outcomes: Adverse Effects

**FIGURE 5:**
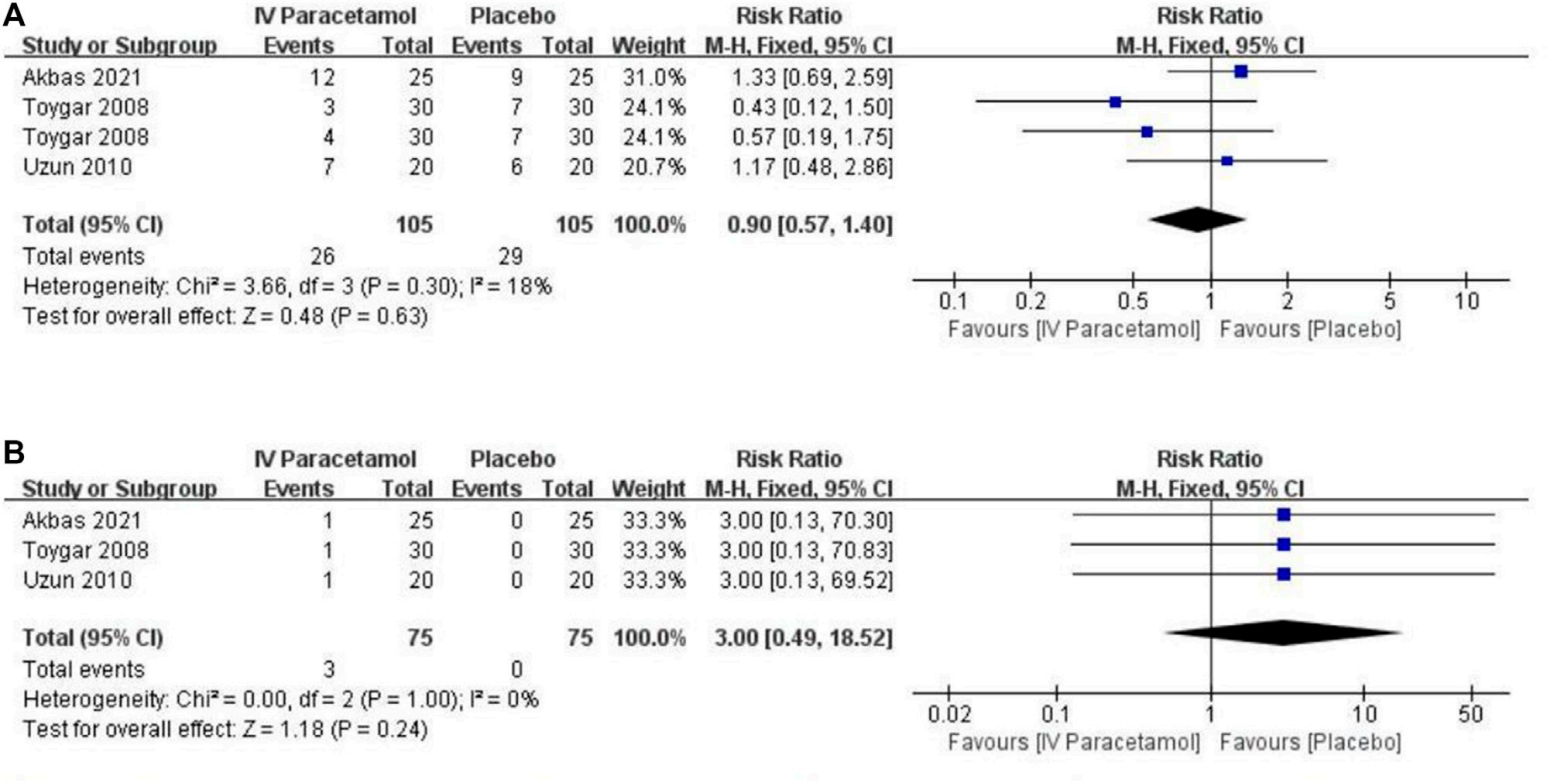
Forest plot showing the incidence of **(A)** nausea and vomiting and **(B)** urinary retention. IV, intravenous. CI, confidence interval.

For IV paracetamol versus placebo, three studies provided data about adverse effects that were permissive to statistical pooling (Toygar et al., 2008; Uzun et al., 2010; Akbas et al., 2021). There was no statistically significant difference between the two groups in postoperative nausea and vomiting (PONV) [RR, 0.90 (95%CI, 0.57–1.40), *p* = 0.63, I^2^ = 18%) and urinary retention [RR, 3.00 (95%CI, 0.49–18.52), *p* = 0.24, I^2^ = 0%), with no heterogeneity between the groups (Figure 5). The GRADE of evidence was rated as low for PONV and moderate for urinary retention (Supplementary Table S2).

## Discussion

The systematic review and meta-analysis were conducted to demonstrate the analgesic efficacy of IV paracetamol in the postoperative period in patients undergoing lumbar disc surgery. Our review analyzed the pooled data from five RCTs and found that patients who received IV paracetamol not only required a significant reduction in morphine consumption, but also had a notable reduction in the pain scores. However, this study did not show any significant difference in the incidence of PONV and urinary retention. The quality of evidence was adjudged as low to moderate because of significant heterogeneity in the pooled data.

According to this review and the meta-analysis, in patients who received IV paracetamol, the cumulative opioid consumption was reduced at 24 h postoperatively with an overall reduction of 10.61 mg compared with placebo. The result was in agreement with previous meta-analyses investigating the effects of intravenous paracetamol on opioid consumption and postoperative pain after total hip or knee arthroplasty (Liang et al., 2017; Guo et al., 2018), bariatric surgery (Lee et al., 2019; Ghaffarpasand et al., 2020), and caesarean (Ng et al., 2019). Paracetamol has been theorized to decrease opioid consumption because of enhancing pain management (Xuan et al., 2021). Based on our analysis, the preoperative administration of paracetamol seemed to have preemptive analgesic effects on patients undergoing lumbar disc surgery (*p* < 0.00001), which was in concordance with the meta-analysis of Xuan et al. (2021) that indicated preemptive IV paracetamol administration significantly reduces opioid consumption following general anesthesia. But we had no idea about the most optimal time of IV paracetamol used. Thus, the performance of more well-conducted RCTs comparing different times of IV paracetamol used on postoperative analgesia is needed. Kurtovic et al. (2017) compared the postoperative analgesic efficacy of intravenous paracetamol administered intermittently and through a patient-controlled analgesia (PCA) pump following lumbar discectomy. They found an interesting result, that patients in both groups had satisfactory pain control results. Moreover, postoperative use of paracetamol analgesia through a PCA pump achieved better pain management versus intermittent paracetamol analgesia after lumbar discectomy (*p* < 0.05). This may provide us with a more suitable analgesic strategy for clinical practice.

We also found that patients receiving single-dose (*p* = 0.0006) and repeated-dose (*p* = 0.003) IV paracetamol on the postoperative day had lower morphine consumption after lumbar disc surgery when compared with placebo. However, in a retrospective cohort study, Morwald et al. (2018) found that postoperative opioid consumption was higher in patients who received a single dose of intravenous paracetamol on the day of surgery. Some reasons could explain this result. First, the pain intensity was so severe that patients had already taken enough opioids but could not completely relieve the pain (Mörwald et al., 2018). Thus, they used intravenous paracetamol as a substitute. Second, these patients in the study often already suffer from preexisting chronic pain treated with conventional analgesics or opioids that may alter the pain perception of these patients (Roullet et al., 2009; Jørgensen et al., 2018). Third, there was no unified standard for administrating intravenous paracetamol, which may be affected by factors such as the patients’ propensities for increased postoperative pain, subjective pain thresholds or hospital policies, and personal preferences of the doctors. Fourth, it was a retrospective cohort study and all the data were observational. Therefore, it was not possible to establish a definitive causal relationship. Moreover, intravenous paracetamol was associated with minimal changes in the length of hospital stay and hospitalization costs. But there was no study on comparing the opioid-sparing effect between different dosages of paracetamol in lumbar discectomy. Further study about the association between the dose of IV paracetamol and opioid consumption would be necessary.

The comparison between intravenous paracetamol and oral paracetamol is a concerning issue. Although there is no research on comparing intravenous paracetamol with oral paracetamol in lumbar surgery, Hansen et al. (2017) conducted a comparative analysis among spine surgery patients with postoperative pain management including intravenous versus oral paracetamol. The study demonstrated that intravenous paracetamol would incur higher specialist and anesthesia costs, however, intravenous paracetamol produced less cumulative hospitalization cost than oral paracetamol (with difference - $1175; 95% CI: - $1611 to - $739; *p* < 0.0001) because it reduced the use of analgesic drugs (with difference -13 mg; 95% CI: -14 mg to -12 mg; *p* < 0.0001) and shortened the length of hospitalization (with difference - 0.68 days; 95% CI: -0.76 to -0.59; *p* < 0.0001). However, there were many limitations in this retrospective cohort study, such as unequal population baselines, many confounding factors of perioperative analgesia schemes, and failure to unify surgical types, which made the external validity of this conclusion limited. In a review and trial sequential analysis of the perioperative comparison between intravenous and oral paracetamol by Mallama et al. (2021), 14 RCTs were included. The types of surgery included joint replacement, cesarean section, endoscopic sinus surgery, oral surgery, hernia surgery, and so on. The analysis showed that intravenous paracetamol reduced the postoperative pain scores by 0–2 h compared with oral paracetamol. But there was no significant difference between the two groups in pain scores during other times, opioid consumption, time to first analgesic request, and postoperative adverse effects. Furthermore, intravenous paracetamol could significantly increase the cost by about $ 47,498 per annum. Of course, there were still some limitations in this study. For example, the quality of the included studies was variable. Secondly, converting the data for analysis instead of directly using the original data may lower the accuracy of the results. The cost analysis was limited to hospitals in the Netherlands. Therefore, more high-quality randomized controlled studies are needed to know whether it is worth the extra cost to use intravenous paracetamol to control pain.

In this meta-analysis, we demonstrated that IV paracetamol showed significantly lower pain scores than the control group, especially in the postoperative 1 h (MD, −2.37), 2 h (MD, −3.17), 12 h (MD, −0.96), and 24 h (MD, −0.97). The same result was also found in the meta-analysis of Ghaffarpasand et al. (2020), which suggested that intravenous paracetamol could decrease postoperatively visual analog scale (VAS) scores in craniotomy surgery. Although the minimal clinically important difference in VAS scores that would signify a clinically important improvement or deterioration in patients with acute pain after surgery has been defined as 9.9 mm (rounded to 0.99 when scored with NRS scores) (Myles et al., 2017), pain scores were inevitably inaccurate because of their subjective nature (Korgvee et al., 2021). In our opinion, IV paracetamol was effective in postoperative analgesia after lumbar disc surgery, especially in the early postoperative period.

Despite the significant reduction in postoperative opioid consumption, we did not find the incidences of opioid-related side effects such as PONV and urinary retention decreased. This is in accordance with the results of a review that investigated the effects of intravenous paracetamol for acute postoperative pain in adults or children (McNicol et al., 2016). Tzortzopoulou et al. also demonstrated that participants receiving intravenous paracetamol required fewer opioids than those receiving the placebo without a reduction in opioid-induced adverse events (Tzortzopoulou et al., 2011). A possible explanation was that many opioid-related adverse effects may be intensified by the neural reflexes caused by surgical stress (Sinatra et al., 2005), thus we were not sure that decreased opioid consumption could lower the risk of the side effects. Also, differences in intraoperative opioid dosing and postoperative analgesic regimen, amount, and type of anesthetics and antiemetics used, timing of IV paracetamol administration, and patient characteristics might have an influence on the incidence of adverse effects (Zayed et al., 2021). Therefore, further studies are needed to make definite recommendations as to whether IV paracetamol might be used to reduce the risks of PONV and urinary retention after lumbar disc surgery.

## Limitations

There are several limitations to this meta-analysis. First, the sample size was low in the included trials, which led to the heterogeneity of this meta-analysis to some extent. However, we conducted random-effects models and subgroup analyses to investigate the high heterogeneity, and the required data size for the primary outcome was found to be absolutely adequate. Second, it was inevitable that there were variations in perioperative analgesic regimens in different trials. But most of the included studies used opioids as intraoperative analgesic medication and morphine as postoperative analgesic medication, which could decrease the bias in our meta-analysis. Third, paracetamol was administered in different doses, but the number of included studies was limited, thus, there was a limited strength of the evidence on the subgroup effect of the doses of IV paracetamol used. We might need more research to explore the effects of different doses of IV paracetamol used on postoperative analgesia. Finally, some included studies calculated incorrect descriptive statistics. We tried to contact the authors of the included studies by email to access the original data. Unfortunately, we have not received a reply so far. We acknowledged that it is a defect of the meta-analysis and that we can only summarize and analyze the secondary data, but not guarantee the correctness of the secondary data. However, we believe that the results of the meta-analysis can represent the trend of secondary data to a certain extent.

## Conclusion

The results of this meta-analysis suggested IV paracetamol might be a valuable medication to reduce opioid consumption and improve postoperative analgesia in patients undergoing lumbar disc surgery. The overall GRADE quality of the evidence was rated as low to moderate. However, additional better-designed and larger studies are necessary to be able to make more definite recommendations, especially on the different doses of IV paracetamol used.

## Data Availability

The original contributions presented in the study are included in the article/Supplementary Material, further inquiries can be directed to the corresponding author.
