# Supplementary material for: Effect of Intravenous Paracetamol on Opioid Consumption in Multimodal Analgesia After Lumbar Disc Surgery: A Meta-Analysis of Randomized Controlled Trials
Source: Front Pharmacol. 2022 May 23;13:860106. doi: 10.3389/fphar.2022.860106 (PMC9168366; doi:10.3389/fphar.2022.860106)
Supplement: Supplementary file 1 [file DataSheet2.PDF]

## Table 2 Summary of findings

## IV paracetamol compared to placebo for lumbar disc surgery

**Patient or population:** patients undergoing lumbar disc surgery

**Settings:** Systematic review and meta-analysis

**Intervention:** IV paracetamol

**Comparison:** Placebo

| Outcomes | Illustrative comparative risks* (95% CI) | | Relative effect (95% CI) | No of Participants (studies) | Quality of the evidence (GRADE) | Comments |
|---|---|---|---|---|---|---|
| | Risk with control | Risk with Paracetamol | | | | |
| **Morphine consumption at 24 h postoperatively** assessed with: morphine (mg) Follow-up: mean 1 days | | MD **10.61 mg lower** (16.00 lower to 5.22 lower) | - | 271 (5 RCTs) | ⊕⊕⊕⊝ **moderate**[a] | IV paracetamol reduces morphine consumption at 24 h postoperatively. |
| **Postoperative pain scores at 1 h** assessed with: score scale from 0 to 10 Follow-up: mean 1 days | | MD **2.37 lower** (3.81 lower to 0.94 lower) | - | 142 (2 studies) | ⊕⊕⊝ ⊝ **moderate**[a] | IV paracetamol results in significant decrease in pain scores at 1 h postoperatively. |
| **Postoperative pain scores at 2 h** assessed with: score scale from 0 to 10 Follow-up: mean 1 days | | MD **3.17 lower** (3.85 lower to 2.48 lower) | - | 90 (1 studiy) | ⊕⊕⊝ ⊝ **moderate**[a] | IV paracetamol results in significant decrease in pain scores at 2 h postoperatively. |
| **Postoperative pain scores at 6 h** assessed with: score scale from 0 to 10 Follow-up: mean 1 days | | MD **1.75 lower** (3.10 lower to 0.40 lower) | - | 142 (2 studies) | ⊕⊕⊝ ⊝ **moderate**[a] | IV paracetamol results in significant difference in pain scores at 6 h postoperatively. |

| | | | | | | |
|---|---|---|---|---|---|---|
| **Postoperative pain scores at 12 h**<br>assessed with: score scale from 0 to 10<br>Follow-up: mean 1 days | | MD **0.96 lower**<br>(1.77 lower to 0.15 lower) | - | 142<br>(2 studies) | ⊕⊕⊕⊝<br>**moderate**[b] | IV paracetamol results in significant decrease in pain scores at 12 h postoperatively. |
| **Postoperative pain scores at 24 h**<br>assessed with: score scale from 0 to 10<br>Follow-up: mean 1 days | | MD **0.97 lower**<br>(1.67 lower to 0.27 lower) | - | 142<br>(2 studies) | ⊕⊕⊝⊝<br>**moderate**[a] | IV paracetamol results in significant decrease in pain scores at 24 h postoperatively. |
| **Postoperative nausea and vomiting (PONV)**<br>assessed with: incidence<br>Follow-up: mean 1 days | **276 per 1000** | **249 per 1000**<br>(157 to 387) | **RR 0.90**<br>(0.57 to 1.40) | 180<br>(3 studies) | ⊕⊕⊝⊝<br>**low**[a,b] | IV paracetamol may result in no difference in PONV |
| **Postoperative urinary retention**<br>assessed with: incidence<br>Follow-up: mean 1 days | **0 per 1000** | **0 per 1000**<br>(0 to 0) | **RR 3.00**<br>(0.49 to 18.52) | 150<br>(3 studies) | ⊕⊕⊕⊝<br>**moderate**[a] | IV paracetamol may result in no difference in urinary retention |

*The basis for the **assumed risk** (e.g. the median control group risk across studies) is provided in footnotes.

The **corresponding risk** (and its 95% confidence interval) is based on the assumed risk in the comparison group and the **relative effect** of the intervention (and its 95% CI).

**CI:** Confidence interval; **MD**: Mean difference; **RR:** Risk ratio; **RCT:** randomized controlled trial; **IV:** intravenous.

**GRADE Working Group grades of evidence**

**High quality:** Further research is very unlikely to change our confidence in the estimate of effect.

**Moderate quality:** Further research is likely to have an important impact on our confidence in the estimate of effect and may change the estimate.

**Low quality:** Further research is very likely to have an important impact on our confidence in the estimate of effect and is likely to change the estimate.

**Very low quality:** We are very uncertain about the estimate.

**Explanations**

a. risks of bias were existed in results

b. results are not completely consistent
