# Supplementary figures and images for: Effect of Intravenous Paracetamol on Opioid Consumption in Multimodal Analgesia After Lumbar Disc Surgery: A Meta-Analysis of Randomized Controlled Trials
Source: Front Pharmacol. 2022 May 23;13:860106. doi: 10.3389/fphar.2022.860106 (PMC9168366; doi:10.3389/fphar.2022.860106)

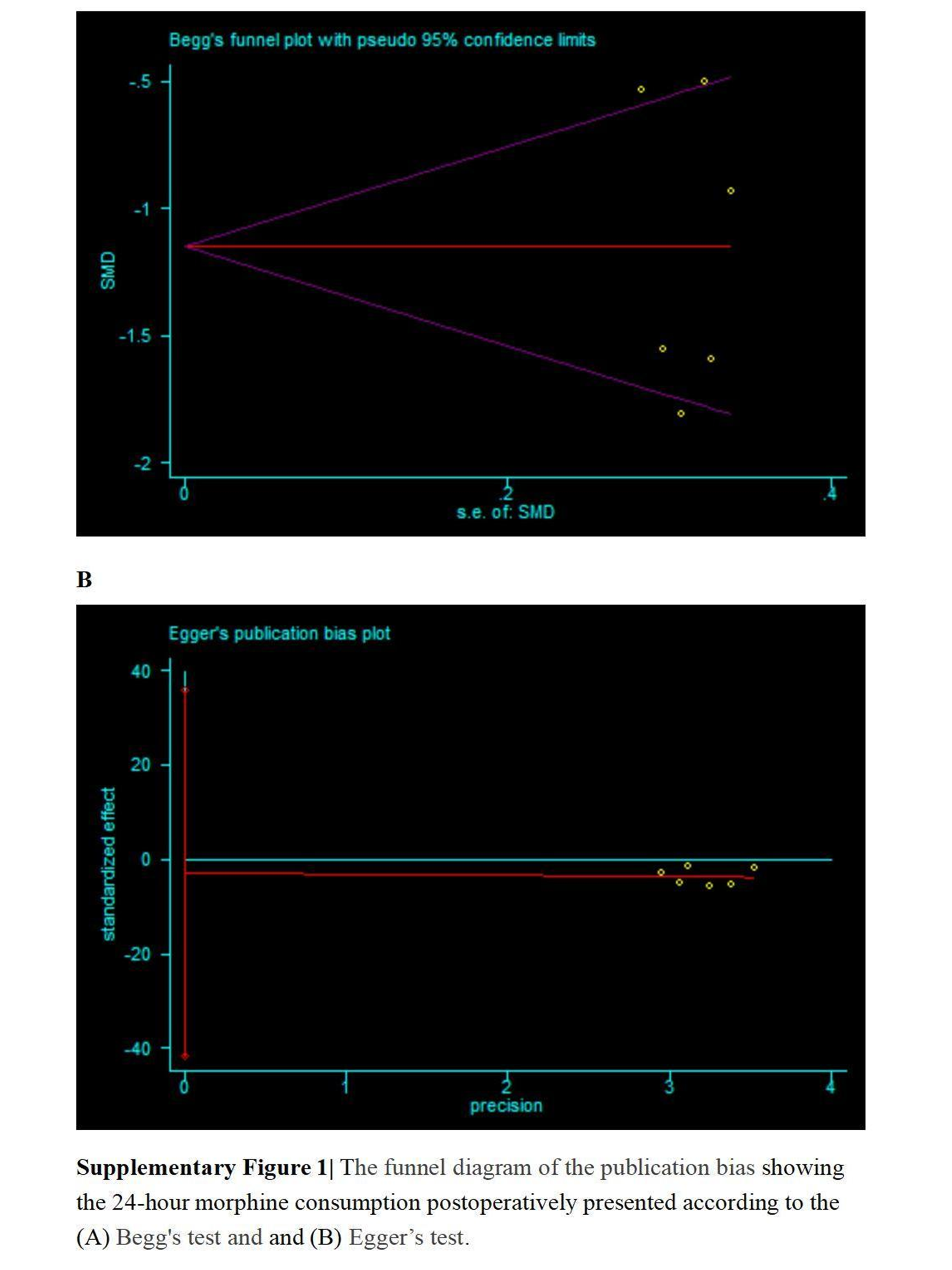

Supplement: Supplementary file 2 [file Image1.TIF]
