# Supplementary material for: Effect of Intravenous Paracetamol on Opioid Consumption in Multimodal Analgesia After Lumbar Disc Surgery: A Meta-Analysis of Randomized Controlled Trials
Source: Front Pharmacol. 2022 May 23;13:860106. doi: 10.3389/fphar.2022.860106 (PMC9168366; doi:10.3389/fphar.2022.860106)
Supplement: Supplementary file 3 [file DataSheet1.PDF]

TABLE S1. Search strategy

Cochrane central

#1 MeSH descriptor: [Acetominophen] explode all trees

#2 (IV):ti,ab,kw

#3 (intravenous):ti,ab,kw

#4 #2 OR #3

#5(Lumbar):ti,ab,kw

#6 #1 AND #4 AND #5

Pubmed

1. Acetaminophen[Mesh Term]
2. Acetominophen[Title/Abstract]
3. Hydroxyacetanilide[Title/Abstract]
4. APAP[Title/Abstract]
5. p-Acetamidophenol[Title/Abstract]
6. p-Hydroxyacetanilide[Title/Abstract]
7. Paracetamol[Title/Abstract]
8. N-(4-Hydroxyphenyl)acetanilide[Title/Abstract]
9. Acetamidophenol[Title/Abstract]
10. N-Acetyl-p-aminophenol[Title/Abstract]
11. Acephen[Title/Abstract]
12. Acetaco[Title/Abstract]
13. Tylenol[Title/Abstract]
14. Anacin-3[Title/Abstract]
15. Anacin 3[Title/Abstract]
16. Anacin3[Title/Abstract]
17. Datril[Title/Abstract]
18. Panadol[Title/Abstract]
19. Acamol[Title/Abstract]
20. Algotropyl[Title/Abstract]
21. 1 OR 2 OR 3 OR 4 OR 5 OR 6 OR 7 OR 8 OR 9 OR 10 OR 11 OR 12 OR 13 OR 14 OR 15 OR 16 OR 17 OR 18 OR 19 OR 20
22. IV[Title/Abstract]
23. Intravenous[Title/Abstract]
24. 22 OR 23
25. lumbar[Title/Abstract]
26. randomized controlled trial[Publication Type]
27. controlled clinical trial[Publication Type]
28. randomized[Title/Abstract]
29. placebo[Title/Abstract]
30. randomly[Title/Abstract]
31. trial[Title/Abstract]
32. groups[Title/Abstract]
33. 25 OR 26 OR 27 OR 28 OR 29 OR 30 OR 31 OR 32
34. 21 AND 24 AND 33

Embase

#1 'acetaminophen'/exp OR hydroxyacetanilide:ti,ab,kw OR apap:ti,ab,kw OR 'p acetamidophenol':ti,ab,kw OR 'p hydroxyacetanilide':ti,ab,kw OR paracetamol:ti,ab,kw OR (n-:ti,ab,kw AND '4 hydroxyphenyl':ti,ab,kw AND acetanilide:ti,ab,kw) OR acetamidophenol:ti,ab,kw OR 'n acetyl p aminophenol':ti,ab,kw OR acephen:ti,ab,kw OR acetaco:ti,ab,kw OR tylenol:ti,ab,kw OR 'anacin 3':ti,ab,kw OR anacin3:ti,ab,kw OR datril:ti,ab,kw OR panadol:ti,ab,kw OR acamol:ti,ab,kw OR algotropyl:ti,ab,kw

#2 IV:ti,ab,kw OR intravenous:ti,ab,kw

#3 lumbar:ti,ab,kw

#3 #1 AND #2 AND #3

Web of science

1.TS=Acetominophen
2. TS=Hydroxyacetanilide
3. TS=APAP
4. TS=p-Acetamidophenol
5. TS=p-Hydroxyacetanilide
6. TS=Paracetamol
7. TS=N-(4-Hydroxyphenyl)acetanilide
8. TS=Acetamidophenol
9. TS=N-Acetyl-p-aminophenol
10. TS=Acephen
11. TS=Acetaco
12. TS=Tylenol
13. TS=Anacin-3
14. TS=Anacin 3
15. TS=Anacin3
16. TS=Datril
17. TS=Panadol
18. TS=Acamol
19. TS=Algotropyl
20.1 OR 2 OR 3 OR 4 OR 5 OR 6 OR 7 OR 8 OR 9 OR 10 OR 11 OR 12 OR 13 OR 14 OR 15 OR 16 OR 17 OR 18 OR 19
21.TS=IV
22.TS=intravenous
23.21 OR 22
24.TS=lumbar
22. 20 AND 23 AND 24

Scopus

((TITLE-ABS-KEY (lumbar AND surgery) AND TITLE-ABS-KEY (paracetamol OR acetominophen) AND TITLE-ABS-KEY (IV OR intravenous)) AND (LIMIT-TO DOCTYPE , "ar"))
